# Supplementary material for: Identification of Freezing-Responsive microRNAs and Their Targets in Chinese Jujube by Small RNA and Degradome Sequencing
Source: Int J Mol Sci. 2026 Sep 4;27(17):7912. doi: 10.3390/ijms27177912 (PMC13566890; doi:10.3390/ijms27177912)
Supplement: Supplementary file 1 [file ijms-27-07912-s001.zip › Supplementary Material S2/novel_miR_162.pdf]

Provisional ID	:	novel_miR_162
Score total	:	3510.9
Score for star read(s)	:	3.9
Score for read counts	:	3505.2
Score for mfe	:	0.2
Score for randfold	:	1.6
Score for cons. seed	:	
Total read count	:	6887
Mature read count	:	6638
Loop read count	:	0
Star read count	:	249

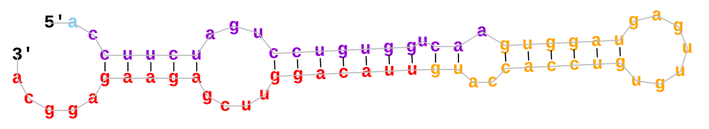

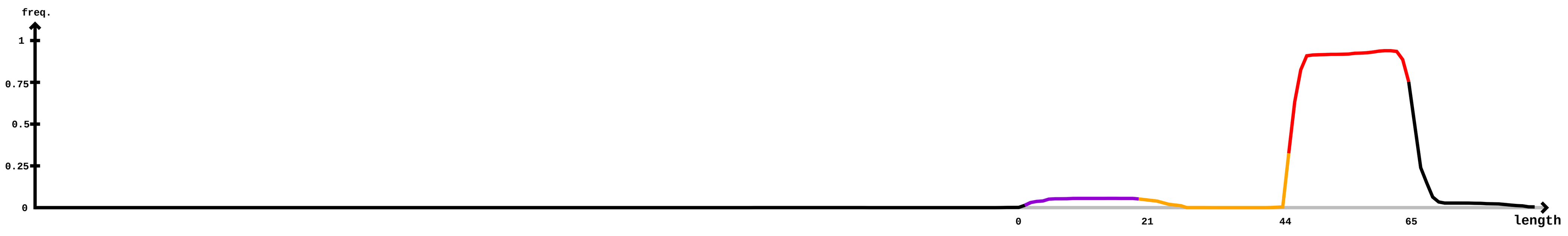

Star Mature

5'- aguuuuaggucagagaguagaacuuaaaaaacaauaagauaagggcaaaauauuggucuaagugaccaauuuuuuggaguuuguacuacauaaugagaccuuucgguaauuuuuauaaaaggagucaucauauaggacacaagaaaaucuaccagguggcccaaccuucuaguccuguggucaaguggaugaguuguguccaccauguuacagguucgagaagaggcaggcacaaacaaaacaaacaacc -3'	obs		
aguuuuaggucagagaguagaacuuaaaaaacaauaagauaagggcaaaauauuggucuaagugaccaauuuuuuggaguuuguacuacauaaugagaccuuucgguaauuuuuauaaaaggagucaucauauaggacacaagaaaaucuaccagguggcccaaccuucuaguccuguggucaaguggaugaguuguguccaccauguuacagguucgagaagaggcaggcacaaacaaaacaaacaacc	exp		
..(((((((((........).)))))))))...............(((((.(((((((.....))))))).)))))..((((((.(((.((.((((..(((((..((((...)))).)))))..)))).)).)))..(((..(.........)..)))(((...((((((...(((((((.((.((((((.......))))))..)))))))))....))))).)...))).........))))))....	reads	mm	sample
aaggagucaucauauagga	2	0	JT-3
cccaaccuucuaguccugug	2	0	LT-3
ccaaccuucuaguccugugguca	1	0	LT-3
ccaaccuucuaguccuguggucaag	4	0	LCK-3
caaccuucuaguccugugguc	2	0	JCK-3
aaccuucuaguccugugguca	1	0	LT-3
accuucuaguccugugguc	1	0	JCK-1
accuucuaguccugugguc	1	0	JCK-3
accuucuaguccugugguc	1	0	JCK-2
accuucuaguccuguggucaa	1	0	JCK-2
accuucuaguccuguggucaa	1	0	LT-3
accuucuaguccuguggucaagu	20	0	LCK-1
accuucuaguccuguggucaagu	27	0	LCK-2
accuucuaguccuguggucaagug	19	0	LCK-2
accuucuaguccuguggucaagug	20	0	LCK-1
ccuucuaguccugugguc	2	0	JCK-1
ccuucuaguccugugguc	2	0	JCK-3
ccuucuaguccugugguc	5	0	LCK-1
ccuucuaguccugugguc	6	0	LCK-2
ccuucuaguccugugguc	3	0	LT-3
ccuucuaguccugugguc	2	0	LT-1
ccuucuaguccugugguca	1	0	LT-1
ccuucuaguccugugguca	1	0	LT-3
ccuucuaguccugugguca	1	0	LCK-2
ccuucuaguccugugguca	1	0	JCK-1
ccuucuaguccuguggucaa	28	0	LCK-2
ccuucuaguccuguggucaa	28	0	LCK-1
ccuucuaguccuguggucCa	1	1	LCK-1
ccuucuaguccuguggucaag	4	0	LT-2
ccuucuaguccuguggucaag	9	0	LCK-3
ccuucuaguccuguggucaagu	2	0	JCK-1
ccuucuaguccuguggucaagu	1	0	JCK-3
ccuucuaguccuguggucaagu	3	0	JCK-2

```
                                                                                                                                                                    Star                                        Mature
aguuuuaggucagagaguagaacuuaaaaaacaauaagauaagggcaaaauauuggucuaagugaccaauuuuuuggaguuuguacuacauaaugagaccuuucgguaauuuuuauaaaaggagucaucauauaggacacaagaaaaucuaccagguggcccaaccuucuaguccuguggucaaguggaugaguuguguccaccauguuacagguucgagaagaggcaggcacaaacaaaacaaacaacc
....................................................................................................................................................................ccuucuaguccuguggucaagug...............................................................   2   0   JT-2
....................................................................................................................................................................ccuucuaguccuguggucaagug...............................................................   1   0   JT-3
....................................................................................................................................................................ccuucuaguccuguggucaagugga.............................................................   3   0   JT-2
....................................................................................................................................................................ccuucuaguccuguggucaagugga.............................................................   5   0   JT-3
....................................................................................................................................................................ccuucuaguccuguggucaagugga.............................................................   1   0   LT-2
....................................................................................................................................................................ccuucuaguccuguggucaaguggau...........................................................   5   0   LT-3
....................................................................................................................................................................ccuucuaguccuguggucaaguggau...........................................................   2   0   LT-2
....................................................................................................................................................................ccuucuaguccuguggucaaguggau...........................................................   3   0   LT-1
.....................................................................................................................................................................cuucuaguccuguggucU...................................................................   1   1   LT-3
.....................................................................................................................................................................cuucuaguccugugguca...................................................................   2   0   JCK-3
.....................................................................................................................................................................cuucuaguccugugguca...................................................................   1   0   JCK-1
.....................................................................................................................................................................cuucuaguccuguggucaa..................................................................   1   0   LT-1
.....................................................................................................................................................................cuucuaguccuguggucaa..................................................................   1   0   LT-3
.....................................................................................................................................................................cuucuaguccuguggucaag.................................................................   1   0   LT-3
.....................................................................................................................................................................cuucuaguccuguggucaagu................................................................   2   0   LT-3
.....................................................................................................................................................................cuucuaguccuguggucaaUu................................................................   2   1   LT-3
.....................................................................................................................................................................cuucuaguccuguggucaagug...............................................................   2   0   LT-1
.....................................................................................................................................................................cuucuaguccuguggucaaguU...............................................................   1   1   LT-2
.....................................................................................................................................................................cuucuaguccuguggucaagugga.............................................................   1   0   LT-3
.....................................................................................................................................................................cuucuCguccuguggucaaguggau...........................................................   1   1   LT-2
.....................................................................................................................................................................cuucuaguccuguggucaaguggau...........................................................   5   0   JCK-3
.....................................................................................................................................................................cuucuaguccuguggucaaguggau...........................................................   7   0   JCK-2
.....................................................................................................................................................................cuucuaguccuguggucaaguggau...........................................................   1   0   LT-3
.....................................................................................................................................................................cuucuaguccuguggucaaguggau...........................................................   5   0   JCK-1
.....................................................................................................................................................................cuucuaguccuguggucaaguggau...........................................................   13  0   LT-2
......................................................................................................................................................................uucuaguccuguggucaagug...............................................................   4   0   LT-3
......................................................................................................................................................................uucuaguccuguggucaagug...............................................................   4   0   LT-2
......................................................................................................................................................................uucuaguccuguggucaagugg..............................................................   2   0   LT-1
......................................................................................................................................................................uucuaguccuguggucaagugg..............................................................   4   0   LT-3
......................................................................................................................................................................uucuaguccuguggucaagugga.............................................................   2   0   JCK-1
......................................................................................................................................................................uucuaguccuguggucaaguggau............................................................   2   0   LT-1
.......................................................................................................................................................................ucuaguccuguggucaagu................................................................   2   0   LCK-3
.......................................................................................................................................................................ucuaguccuguggucaaUu................................................................   1   1   LT-3
.......................................................................................................................................................................ucuaguccuguggucaagu................................................................   1   0   LT-2
.......................................................................................................................................................................ucuaguccuguggucaagu................................................................   2   0   LT-3
.......................................................................................................................................................................ucuaguccuguggucaagug...............................................................   1   0   LT-2
.......................................................................................................................................................................ucuaguccuguggucaaguU...............................................................   2   1   JCK-2
.......................................................................................................................................................................ucuaguccuguggucaaguU...............................................................   1   1   LT-3
.......................................................................................................................................................................ucuaguccuguggucaagug...............................................................   1   0   LT-1
.......................................................................................................................................................................ucuaguccuguggucaagug...............................................................   3   0   LCK-3
.......................................................................................................................................................................ucuaguccuguggucaagug...............................................................   2   0   LT-3
.......................................................................................................................................................................ucuaguccuguggucaaguU...............................................................   4   1   JCK-3
.......................................................................................................................................................................ucuaguccuguggucaaguU...............................................................   1   1   LT-2
.......................................................................................................................................................................ucuaguccuguggucaagugg..............................................................   1   0   JCK-1
.......................................................................................................................................................................ucuaguccuguggucaagugg..............................................................   4   0   LT-1
.......................................................................................................................................................................ucuaguccuguggucaagugg..............................................................   5   0   JCK-3
.......................................................................................................................................................................ucuaguccuguggucaagugg..............................................................   2   0   JCK-2
.......................................................................................................................................................................ucuaguccuguggucaagugg..............................................................   6   0   LT-3
.......................................................................................................................................................................ucuaguccuguggucaagugg..............................................................   5   0   LCK-3
.......................................................................................................................................................................ucuaguccuguggucaaguggU.............................................................   2   1   LCK-3
.......................................................................................................................................................................ucuCguccuguggucaaguggau............................................................   1   1   LT-2
.......................................................................................................................................................................ucuaguccuguggucaaguggau............................................................   8   0   LCK-3
.......................................................................................................................................................................ucuaguccuguggucaaguggau............................................................   1   0   LT-3
.......................................................................................................................................................................ucuaguccuguggucaaguggau............................................................   7   0   JCK-1
.......................................................................................................................................................................ucuaguccuguggucaaguggau............................................................   4   0   LT-2
.......................................................................................................................................................................ucuaguccuguggucaaguggau............................................................   2   0   JCK-3
.......................................................................................................................................................................ucuaguccuguggucaaguggau............................................................   2   0   LT-1
.......................................................................................................................................................................ucuaguccuguggucaaguggau............................................................   9   0   JCK-2
........................................................................................................................................................................cuaguccuguggucaCgu................................................................   1   1   LT-2
........................................................................................................................................................................cuaguccuguggucaagu................................................................   2   0   LT-2
........................................................................................................................................................................cuaguccuguggucaagu................................................................   3   0   LT-3
........................................................................................................................................................................cuaguccuguggucaagu................................................................   1   0   LT-1
........................................................................................................................................................................cuaguccuguggucaagug...............................................................   1   0   LT-3
........................................................................................................................................................................cuaguccuguggucaagug...............................................................   1   0   LT-1
........................................................................................................................................................................cuaguccuguggucaagugg..............................................................   1   0   JCK-1
........................................................................................................................................................................cuaguccuguggucaaguggau............................................................   2   0   LT-3
........................................................................................................................................................................cuaguccuguggucaaguggau............................................................   1   0   JCK-1
........................................................................................................................................................................cuaguccuguggucaaguggau............................................................   2   0   JCK-2
.........................................................................................................................................................................uaguccuguggucaagugg..............................................................   1   0   LT-3
..........................................................................................................................................................................aguccuguggucaagugg..............................................................   1   0   JCK-2
```

Star Mature

aguuuuaggucagagaguagaacuuaaaaaacaauaagauaagggcaaaauauuggucuaagugaccaauuuuuuggaguuuguacuacauaaugagaccuuucgguaauuuuuauaaaaggagucaucauauaggacacaagaaaaucuaccagguggcccaaccuucuaguccuguggucaaguggaugaguuguguccaccauguuacagguucgagaagaggcaggcacaaacaaaacaaacaacc

aguccuguggucaagugga	1	0	LT-3
guccuguggucaagugga	4	0	JT-2
guccuguggucaagugga	10	0	JT-3
uccuguggucaaguggauCagu	1	1	LCK-1
uggucaaguggaugaguu	1	0	LT-3
auCuuacagguucgagaag	1	1	LT-3
auCuuacagguucgagaagagg	1	1	LCK-1
auCuuacagguucgagaagaggc	1	1	JT-3
auNuuacagguucgagaagaggcagg	1	1	LT-2
auCuuacagguucgagaagaggcaggc	1	1	JCK-2
uCuuacagguucgagaagag	1	1	LT-2
uCuuacagguucgagaagagg	2	1	LT-2
uCuuacagguucgagaagagg	1	1	LCK-3
uCuuacagguucgagaagagg	1	1	LCK-1
uCuuacagguucgagaagaggc	1	1	LT-3
uCuuacagguucgagaagaggca	1	1	LCK-3
uCuuacagguucgagaagaggca	2	1	JT-3
uCuuacagguucgagaagaggca	2	1	LT-2
uCuuacagguucgagaagaggcaggc	1	1	LT-2
Cuuacagguucgagaaga	1	1	LCK-1
Cuuacagguucgagaagagg	2	1	LT-2
Cuuacagguucgagaagaggca	1	1	LT-1
guuacagguucgagaagaggca	1	0	LCK-3
Uuuacagguucgagaagaggca	6	1	LT-2
Cuuacagguucgagaagaggcag	1	1	LCK-3
Cuuacagguucgagaagaggcagg	1	1	JT-1
Cuuacagguucgagaagaggcaggc	1	1	LCK-3
uuacagguucgagaagag	7	0	JT-3
uuacagguucgagaagag	9	0	JT-2
uuacagguucgagaagag	5	0	LT-2
uuacagguucgagaagag	6	0	LT-1
uuacagguucgagaagag	2	0	LCK-3
uuacagguucgagaagag	1	0	LT-3
uuacagguucgagaagagg	6	0	JCK-3
uuacagguucgagaagagg	21	0	LT-1
uuacagguucgagaagagg	4	0	JCK-1
uuacagguucgagaagagg	3	0	JCK-2
uuacagguucgagaagagU	1	1	LT-2
uuacagguucgagaagagg	44	0	JT-3
uuacagguucgagaagagA	9	1	LCK-3
uuacagguucgagaagagg	8	0	LCK-3
uuacagguucgagaagagg	35	0	LT-3
uuacagguucgagaagagg	30	0	JT-2
uuacCgguucgagaagagg	1	1	LCK-3
uuacagguucgagaagagg	16	0	LT-2
uuacCgguucgagaagagg	1	1	LT-3
uuacagguucgagaagaggc	25	0	LCK-3
uuacagguucgagaagaggc	12	0	JT-2
uuacagguucgagaagaggc	69	0	LT-1
uuacagguucgagaagaggc	1	0	JCK-3
uuacagguucgagaagaggc	66	0	LT-3
uuacagguucgagaagaggc	6	0	JCK-1
uuacagguucgagaagaggc	11	0	JT-1
uuacagguucgagaagaggc	57	0	LT-2
uuacagguucgagaagaggc	13	0	JT-3
uuNcagguucgagaagaggc	1	1	LT-2
uuacCgguucgagaagaggc	1	1	LT-3
uuacagguucgagaagaggc	7	0	JCK-2
uuacagguucgCgaagaggc	1	1	LT-2
uuacagguucgagaagagAca	1	1	LT-3
uuacagguucgagaagaggca	414	0	LCK-3
uuaAagguucgagaagaggca	1	1	LT-1
uuacCgguucgagaagaggca	1	1	LCK-3
uuacagguucgagaagaggca	13	0	JCK-2
uuacagguucgagaagaggca	23	0	JT-3
Cuacagguucgagaagaggca	1	1	LCK-3
uuacagguucgagaagaAgca	1	1	LT-1
uuacagguucgCgaagaggca	1	1	LCK-2
uuacaAguucgagaagaggca	1	1	LT-2
uuacagguucgCgaagaggca	1	1	LT-2

Star Mature

```
aguuuuaggucagagaguagaacuuaaaaaacaauaagauaagggcaaaauauuggucuaagugaccaauuuuuuggaguuuguacuacauaaugagaccuuucgguaauuuuuauaaaaggagucaucauauaggacacaagaaaaucuaccagguggcccaaccuucuaguccuguggucaaguggaugaguuguguccaccauguuacagguucgagaagaggcaggcacaaacaaaacaaacaacc
...............................................................................................................................................................................................................uuacagAuucgagaagaggca......................   1     1   LCK-3
...............................................................................................................................................................................................................uuacagguucgagaagaggca......................   243   0   LT-2
...............................................................................................................................................................................................................uuacagguucgagaagaggUa......................   1     1   JCK-3
...............................................................................................................................................................................................................uuacagguucgagaagaggca......................   4     0   JCK-3
...............................................................................................................................................................................................................uuacagguucgagaagaggca......................   70    0   LCK-2
...............................................................................................................................................................................................................uuacagguucgagaagaggca......................   83    0   LCK-1
...............................................................................................................................................................................................................uuacagguucgagaagaggca......................   161   0   LT-1
...............................................................................................................................................................................................................uNacagguucgagaagaggca......................   1     1   JT-2
...............................................................................................................................................................................................................uuacagguucgagaagaggca......................   44    0   JT-2
...............................................................................................................................................................................................................uuacCgguucgagaagaggca......................   1     1   LT-2
...............................................................................................................................................................................................................uuNcagguucgagaagaggca......................   1     1   LT-2
...............................................................................................................................................................................................................uuacagguucgagaagaggca......................   15    0   JCK-1
...............................................................................................................................................................................................................uuacagguucgagaagaggca......................   159   0   LT-3
...............................................................................................................................................................................................................uuacagguucgagaagaggcag.....................   28    0   LCK-1
...............................................................................................................................................................................................................uuacCgguucgagaagaggcag.....................   1     1   LT-3
...............................................................................................................................................................................................................uuacagguucgagaagaggcag.....................   43    0   LCK-3
...............................................................................................................................................................................................................uuacagguucgagaagaggcag.....................   14    0   LT-1
...............................................................................................................................................................................................................uuacagguucgagaagaggcag.....................   23    0   LCK-2
...............................................................................................................................................................................................................uuacagguucgagaagaggcag.....................   26    0   JT-3
...............................................................................................................................................................................................................uuacagguucgagaagaggcag.....................   17    0   JT-2
...............................................................................................................................................................................................................uuacagguucgagaagaggcag.....................   16    0   LT-3
...............................................................................................................................................................................................................uuacagguucgagaagaggcag.....................   26    0   LT-2
...............................................................................................................................................................................................................uuacagguucgagaagaggcagg....................   37    0   LT-2
...............................................................................................................................................................................................................uuacagguucgagaagaggcagg....................   2     0   JCK-3
...............................................................................................................................................................................................................uuacagguucgagaagaggcagA....................   1     1   LT-1
...............................................................................................................................................................................................................uuacagguucgagaagaggcagg....................   145   0   LCK-3
...............................................................................................................................................................................................................uuacagguucgagaagaggcagg....................   6     0   JT-1
...............................................................................................................................................................................................................uuacagguucgagaagaggcagU....................   3     1   LT-2
...............................................................................................................................................................................................................uuacagguucgagaagaggcagg....................   5     0   JCK-2
...............................................................................................................................................................................................................uuacagguucgagaagaggcagg....................   44    0   LT-1
...............................................................................................................................................................................................................uuacagguucgagaagaggcagg....................   16    0   JT-3
...............................................................................................................................................................................................................uuacagguucgagaagaggcagg....................   12    0   JT-2
...............................................................................................................................................................................................................uuacagguucgagaagaggcagg....................   2     0   JCK-1
...............................................................................................................................................................................................................uuacagguucgagaagaggcagg....................   28    0   LT-3
...............................................................................................................................................................................................................uuacagguucgagaaUaggcaggc...................   1     1   JCK-1
...............................................................................................................................................................................................................uuacagguucgagaagaggcaggc...................   2     0   JCK-3
...............................................................................................................................................................................................................uuacagguucgagaagaggcaggc...................   1     0   JCK-2
...............................................................................................................................................................................................................uuacagguucgagaagaggcaggc...................   17    0   LT-3
...............................................................................................................................................................................................................uuacagguucgagaagaggcaggc...................   10    0   LCK-3
...............................................................................................................................................................................................................uuacagguucgagaagaggcaggU...................   1     1   LT-3
...............................................................................................................................................................................................................uuacagguucgagaagaggcaggc...................   16    0   LT-2
...............................................................................................................................................................................................................uuacagguucgagaaUaggcaggc...................   3     1   JCK-3
...............................................................................................................................................................................................................uuacagguucgagaaUaggcaggc...................   1     1   JCK-2
...............................................................................................................................................................................................................uuacagguucgagaagaggcaggc...................   17    0   LT-1
...............................................................................................................................................................................................................uuacagguucgCgaagaggcaggca..................   1     1   LCK-3
...............................................................................................................................................................................................................uuacagguucgagaagaggcaggca..................   14    0   LCK-3
...............................................................................................................................................................................................................uuacagguucgagaagaggcaggca..................   3     0   JCK-2
...............................................................................................................................................................................................................uuacagguucgagaagaggcaggca..................   3     0   LT-2
...............................................................................................................................................................................................................uuacagguucgagaagaggcaggca..................   5     0   LT-3
...............................................................................................................................................................................................................uuacagguucgagaagaggcaggca..................   2     0   JT-3
...............................................................................................................................................................................................................uuacagguucgagaagaggcaggca..................   2     0   JT-2
...............................................................................................................................................................................................................uuacagguucgagaagaggcaggca..................   4     0   LT-1
...............................................................................................................................................................................................................uuacagguucgagaagaggcaggca..................   2     0   JCK-1
...............................................................................................................................................................................................................uuacagguucgagaagaggcaggcac.................   3     0   JCK-2
...............................................................................................................................................................................................................uuacagguucgagaagaggcaggcac.................   1     0   LT-1
...............................................................................................................................................................................................................uuacagguucgagaagaggcaggcac.................   2     0   LT-2
................................................................................................................................................................................................................uacagguucgagaagagg........................   1     0   JT-1
................................................................................................................................................................................................................uacagguucgagaagagg........................   28    0   LCK-3
................................................................................................................................................................................................................uacagguucgagaagagg........................   3     0   JCK-3
................................................................................................................................................................................................................uacagguucgagaagagg........................   51    0   LT-2
................................................................................................................................................................................................................uacagguucgagaagagg........................   11    0   JT-3
................................................................................................................................................................................................................uacagguucgagaagagg........................   37    0   LT-3
................................................................................................................................................................................................................uacagguucgagaagagg........................   3     0   JCK-1
................................................................................................................................................................................................................uacagguucgagaagagg........................   7     0   JCK-2
................................................................................................................................................................................................................uacCgguucgagaagagg........................   1     1   LT-3
................................................................................................................................................................................................................uacCgguucgagaagagg........................   1     1   LT-2
................................................................................................................................................................................................................uacagguucgagaagagg........................   51    0   LT-1
................................................................................................................................................................................................................uacagguucgagaagagg........................   5     0   JT-2
................................................................................................................................................................................................................uacagguucgagaagaggc.......................   5     0   JT-3
................................................................................................................................................................................................................uacagguucgagaagaggc.......................   3     0   JCK-3
```

```
                                                                                                                                                                                   Star                Mature
aguuuuaggucagagaguagaacuuaaaaaacaauaagauaagggcaaaauauuggucuaagugaccaauuuuuuggaguuuguacuacauaaugagaccuuucgguaauuuuuauaaaaggagucaucauauaggacacaagaaaaucuaccagguggcccaaccuucuaguccuguggucaaguggaugaguuguguccaccauguuacagguucgagaagaggcaggcacaaacaaaacaaacaacc
................................................................................................................................................................................................................uacagguucgagaagaggc.......................    83    0    LT-1
................................................................................................................................................................................................................uacagguucgagaagaggc.......................    76    0    LCK-3
................................................................................................................................................................................................................uacagguucgagaagaggc.......................    22    0    LCK-2
................................................................................................................................................................................................................uacagguucgagaagaggc.......................    77    0    LT-2
................................................................................................................................................................................................................uacagguucgagaagaggc.......................    77    0    LT-3
................................................................................................................................................................................................................uacagguucgagaagaggc.......................    4     0    JT-2
................................................................................................................................................................................................................uacagguucgagaagaggc.......................    1     0    JCK-1
................................................................................................................................................................................................................uacCgguucgagaagaggc.......................    1     1    LT-2
................................................................................................................................................................................................................uacagguucgagaagaggc.......................    9     0    JCK-2
................................................................................................................................................................................................................uacagguucgagaagaggc.......................    24    0    LCK-1
................................................................................................................................................................................................................uacCgguucgagaagaggc.......................    1     1    LCK-2
................................................................................................................................................................................................................uacagguucgagaagaggca......................    13    0    JT-3
................................................................................................................................................................................................................uacagguucgCgaagaggca......................    1     1    LT-2
................................................................................................................................................................................................................uacCgguucgagaagaggca......................    2     1    LT-2
................................................................................................................................................................................................................uacCgguucgagaagaggca......................    1     1    LT-3
................................................................................................................................................................................................................uacagguucgagaagaggca......................    129   0    LT-1
................................................................................................................................................................................................................uacCgguucgagaagaggca......................    1     1    LCK-3
................................................................................................................................................................................................................uacagguucgagaagaggca......................    184   0    LT-2
................................................................................................................................................................................................................uacagguucgagaagaggca......................    250   0    LCK-3
................................................................................................................................................................................................................uacagguucgagaagaggca......................    24    0    JT-2
................................................................................................................................................................................................................uacagguucgagaagaggca......................    155   0    LT-3
................................................................................................................................................................................................................uacagguucgagaagaggca......................    11    0    JCK-2
................................................................................................................................................................................................................uacagguucgagaagaggca......................    44    0    LCK-2
................................................................................................................................................................................................................uacagguucgagaagaggca......................    10    0    JCK-1
................................................................................................................................................................................................................uacagguucgagaagaggca......................    47    0    LCK-1
................................................................................................................................................................................................................uacagguucgagaagaggca......................    39    0    JT-1
................................................................................................................................................................................................................uacagguucgagaagaggca......................    6     0    JCK-3
................................................................................................................................................................................................................uacagguucgagaagaggcag.....................    1     0    JCK-1
................................................................................................................................................................................................................uacagguucgagaagaggcag.....................    2     0    JCK-3
................................................................................................................................................................................................................uacagguucgagaagaggUag.....................    6     1    JT-2
................................................................................................................................................................................................................uacagguucgagaagaggcag.....................    25    0    LT-2
................................................................................................................................................................................................................uacagguucgCgaagaggcag.....................    1     1    LCK-3
................................................................................................................................................................................................................uacagguucgagaagaggcag.....................    26    0    LCK-2
................................................................................................................................................................................................................uacagguucgagaagaggcag.....................    2     0    JT-3
................................................................................................................................................................................................................uacagguucgagaagaggcag.....................    1     0    JCK-2
................................................................................................................................................................................................................uacagguucgagaCgaggcag.....................    1     1    LCK-1
................................................................................................................................................................................................................uacagguucgagaagaggcag.....................    65    0    LCK-3
................................................................................................................................................................................................................uacagguucgagaagaggUag.....................    3     1    JT-3
................................................................................................................................................................................................................uacagguucgagaagaggcag.....................    2     0    JT-2
................................................................................................................................................................................................................uacCgguucgagaagaggcag.....................    1     1    LCK-3
................................................................................................................................................................................................................uacagguucgagaagaggcag.....................    19    0    LT-3
................................................................................................................................................................................................................uacagguucgagaagaggcag.....................    22    0    LCK-1
................................................................................................................................................................................................................uacagguucgagaagaggcag.....................    22    0    LT-1
................................................................................................................................................................................................................uacagguucgagaagaggcagg....................    2     0    JCK-2
................................................................................................................................................................................................................uacagguucgagaagaggcagg....................    1     0    JCK-1
................................................................................................................................................................................................................uacagguucgagaagaggcagA....................    2     1    LT-3
................................................................................................................................................................................................................uacagguucgagaagaggcagg....................    2     0    JCK-3
................................................................................................................................................................................................................uacagguucgagaagaggcagg....................    17    0    LT-3
................................................................................................................................................................................................................uacagguucgagaagaggcagg....................    34    0    LT-2
................................................................................................................................................................................................................uacagguucgagaagaggcagg....................    20    0    LT-1
................................................................................................................................................................................................................uacagguucgagaagaggcagg....................    51    0    LCK-3
................................................................................................................................................................................................................uacagguucgagaagaggcagg....................    2     0    JT-3
................................................................................................................................................................................................................uacagguucgagaNgaggcaggc...................    1     1    LCK-1
................................................................................................................................................................................................................uacagguucgagaagaggcagUc...................    1     1    LCK-2
................................................................................................................................................................................................................uacagguucgagaaNaggcaggc...................    1     1    LCK-1
................................................................................................................................................................................................................uacagguucgagaagaggcaggc...................    27    0    LT-1
................................................................................................................................................................................................................uacagguucgagaagaggcaggA...................    1     1    LT-3
................................................................................................................................................................................................................uacagguucgagaagaggcaggc...................    69    0    LCK-2
................................................................................................................................................................................................................uacagguucgagaagaggcaggc...................    24    0    LT-3
................................................................................................................................................................................................................uacagguucgagaagaggcaggc...................    32    0    LT-2
................................................................................................................................................................................................................uacagguucgagaagaggcaggc...................    58    0    LCK-1
................................................................................................................................................................................................................uacagguucgagaagaggcaggc...................    3     0    JCK-1
................................................................................................................................................................................................................uacagguucgagaagaggcaggc...................    54    0    LCK-3
................................................................................................................................................................................................................uacagguucgagaagaggcaggc...................    15    0    JCK-2
................................................................................................................................................................................................................uacagguucgagaagaggcaggc...................    5     0    JCK-3
................................................................................................................................................................................................................uacagguucgagaagaggcaggU...................    2     1    JCK-1
................................................................................................................................................................................................................uacagguucgagaagaggcaggca..................    28    0    LCK-1
................................................................................................................................................................................................................uacagguucgagaagaggcaggca..................    10    0    LT-2
................................................................................................................................................................................................................uacagguucgagaagaggcaggca..................    1     0    JCK-2
................................................................................................................................................................................................................uacagguucgagaagaggcaggca..................    6     0    JT-3
```

```
                                                                                                                                                                                                  Star                                                Mature
aguuuuaggucagagaguagaacuuaaaaaacaauaagauaagggcaaaauauuggucuaagugaccaauuuuuuggaguuuguacuacauaaugagaccuuucgguaauuuuuauaaaaggagucaucauauaggacacaagaaaaucuaccagguggcccaaccuucuaguccuguggucaaguggaugaguuguguccaccauguuacagguucgagaagaggcaggcacaaacaaaacaaacaacc
................................................................................................................................................................................................................uacagguucgagaagaggcaggca..................	35	0	LCK-2
................................................................................................................................................................................................................uacagguucgagaagaggcaggca..................	1	0	JT-2
................................................................................................................................................................................................................uacagguucgagaagaggcaggca..................	4	0	LT-3
................................................................................................................................................................................................................uacagguucgagaagaggcaggca..................	5	0	LT-1
................................................................................................................................................................................................................uacagguucgagaagaggcaggca..................	1	0	JCK-3
................................................................................................................................................................................................................uacagguucgagaagaggcaggcac.................	9	0	LCK-3
................................................................................................................................................................................................................uacagguucgagaagaggcaggcac.................	9	0	LT-1
................................................................................................................................................................................................................uacagguucgagaagaggcaggcac.................	3	0	LT-2
................................................................................................................................................................................................................uacagguucgagaagaggcaggcac.................	1	0	LT-3
.................................................................................................................................................................................................................acagguucgagaagaggc.......................	1	0	JCK-1
.................................................................................................................................................................................................................acagguucgagaagaggc.......................	12	0	JT-1
.................................................................................................................................................................................................................acagguucgagaagaggc.......................	41	0	LCK-3
.................................................................................................................................................................................................................acagguucgagaagaggc.......................	61	0	LT-1
.................................................................................................................................................................................................................acagguucgagaagaggc.......................	98	0	LT-3
.................................................................................................................................................................................................................acagguucgagaagaggc.......................	84	0	LT-2
.................................................................................................................................................................................................................acagguucgagaagaggc.......................	1	0	JT-2
.................................................................................................................................................................................................................acagguucgagaagaggc.......................	5	0	JCK-3
.................................................................................................................................................................................................................acagguucgagaagaggc.......................	2	0	JT-3
.................................................................................................................................................................................................................acagguucgagaagaggc.......................	7	0	JCK-2
.................................................................................................................................................................................................................acagguucgagaagaggca......................	5	0	JCK-1
.................................................................................................................................................................................................................acagguucgagaagaggca......................	123	0	LT-2
.................................................................................................................................................................................................................acagguucgCgaagaggca......................	1	1	LCK-2
.................................................................................................................................................................................................................acagguucgagaagaggca......................	8	0	JCK-2
.................................................................................................................................................................................................................acagguucgagaagaggca......................	16	0	JT-3
.................................................................................................................................................................................................................acagguucgagaagaggca......................	3	0	JCK-3
.................................................................................................................................................................................................................acagguucgagaagaggca......................	79	0	LT-1
.................................................................................................................................................................................................................acCgguucgagaagaggca......................	3	1	LT-2
.................................................................................................................................................................................................................acagguucgagaagaggcU......................	1	1	LT-3
.................................................................................................................................................................................................................acNgguucgagaagaggca......................	1	1	LT-2
.................................................................................................................................................................................................................acagguucgagaagaggca......................	99	0	LT-3
.................................................................................................................................................................................................................acagguucgagaagaggca......................	85	0	LCK-2
.................................................................................................................................................................................................................acagguucgagaagaggca......................	122	0	LCK-3
.................................................................................................................................................................................................................acagguucgagaagaggca......................	14	0	JT-2
.................................................................................................................................................................................................................acagguucgagaagaggca......................	79	0	LCK-1
.................................................................................................................................................................................................................aGagguucgagaagaggca......................	1	1	LT-2
.................................................................................................................................................................................................................acagguucgagaagaggcag.....................	14	0	LT-1
.................................................................................................................................................................................................................acagguucgagaagaggcag.....................	17	0	LT-3
.................................................................................................................................................................................................................acagguucgagaagaggcag.....................	1	0	JCK-3
.................................................................................................................................................................................................................acagguucgagaagaggcag.....................	13	0	LT-2
.................................................................................................................................................................................................................acagguucgagaagaggcag.....................	5	0	JCK-1
.................................................................................................................................................................................................................acagguucgagaagaggcag.....................	7	0	JT-2
.................................................................................................................................................................................................................acagguucgagaagaggcag.....................	13	0	JT-1
.................................................................................................................................................................................................................acagguucgagaagaggcag.....................	3	0	JCK-2
.................................................................................................................................................................................................................acagguucgagaagaggcag.....................	41	0	LCK-3
.................................................................................................................................................................................................................acagguucgagaagaggcag.....................	8	0	JT-3
.................................................................................................................................................................................................................acagguucgagaagaggcagg....................	4	0	JCK-2
.................................................................................................................................................................................................................acagguucgagaagaggcagg....................	4	0	JT-3
.................................................................................................................................................................................................................acagguucgagaagaggcagg....................	43	0	LCK-3
.................................................................................................................................................................................................................acagguucgagaagaggcaAg....................	1	1	LCK-3
.................................................................................................................................................................................................................acagguucgagaagaggcagg....................	27	0	LT-2
.................................................................................................................................................................................................................acagguucgagaagaggcagg....................	8	0	LT-3
.................................................................................................................................................................................................................acagguucgagaagaggcagg....................	33	0	LT-1
.................................................................................................................................................................................................................acagguucgagaagaggcagg....................	4	0	JT-2
.................................................................................................................................................................................................................acagguucgagaagaggcagg....................	1	0	JCK-1
.................................................................................................................................................................................................................acagguucgagaagaggcaggc...................	19	0	LT-1
.................................................................................................................................................................................................................acagguucgagaagaggcaggc...................	13	0	LT-3
.................................................................................................................................................................................................................acagguucgagaagaggcaggc...................	51	0	LCK-2
.................................................................................................................................................................................................................acagguucgagaagaggcaggc...................	44	0	LCK-1
.................................................................................................................................................................................................................acagguucgagaagaggcaggc...................	14	0	LCK-3
.................................................................................................................................................................................................................acagguucgagaagaggcaggc...................	16	0	LT-2
.................................................................................................................................................................................................................acagguucgagaagaggcaggca..................	7	0	LT-3
.................................................................................................................................................................................................................acagguucgagaagaggcaggca..................	6	0	LT-1
.................................................................................................................................................................................................................acagguucgagaagaggcaggca..................	1	0	LT-2
.................................................................................................................................................................................................................acagguucgagaagaggcaggcac.................	5	0	LT-2
.................................................................................................................................................................................................................acagguucgagaagaggcaggcac.................	2	0	JCK-3
.................................................................................................................................................................................................................acagguucgagaagaggcaggcac.................	2	0	LT-1
.................................................................................................................................................................................................................Ucagguucgagaagaggcaggcacaaaca............	3	1	JT-3
..................................................................................................................................................................................................................cCgguucgagaagaggca......................	1	1	LT-1
..................................................................................................................................................................................................................cagguucgagaagaggca......................	48	0	LT-3
..................................................................................................................................................................................................................cagguucgagaagaggca......................	18	0	JT-2
```

Star / Mature

aguuuuaggucagagaguagaacuuaaaaaacaauaagauaagggcaaaauauuggucuaagugaccaauuuuuuggaguuuguacuacauaaugagaccuuucgguaauuuuuuauaaaaggagucaucauauaggacacaagaaaaucuaccagguggcccaaccuucuaguccuguggucaaguggaugaguuguguccaccauguuacagguucgagaagaggcaggcacaaacaaaacaaacaacc

cagguucgagaagaggca	59	0	LCK-2
cagguucgagaCgaggca	1	1	LT-2
cagguucgagaagaNgca	1	1	LCK-1
cagguucgagaagaggca	1	0	JCK-2
cagguucgagaagaggca	73	0	LCK-1
cagguucgagaagaggca	14	0	JT-3
cCgguucgagaagaggca	1	1	LT-2
cagguucgagaagaggca	2	0	JCK-3
cagguucgagaagaggca	34	0	LT-1
cagguucgagaagaggca	54	0	LT-2
cagguucgagaagaggca	22	0	LCK-3
cagguucgagaagaggca	2	0	JCK-1
cagguucgagaagaggcag	13	0	JT-2
cagguucgagaagaggcag	6	0	LT-1
cagguucgagaagaggcag	11	0	LT-2
cagguucgagaagaggcag	3	0	LT-3
cagguucgagaagaggcag	1	0	JCK-2
cagguucgagaagaggcag	19	0	JT-3
cagguucgagaagaggcag	8	0	LCK-3
cagguucgagaagaggcagg	5	0	LT-2
cagguucgagaagaggcagg	10	0	JT-2
cagguucgagaagaggcagg	1	0	JCK-1
cagguucgagaagaggcagg	16	0	LT-3
cagguucgagaagaggcagg	9	0	LCK-3
cagguucgagaagaggcagg	2	0	JCK-2
cagguucgagaagaggcagA	1	1	LT-2
cagguucgagaagaggcagg	16	0	JT-3
cagguucgagaagaggcagg	9	0	LT-1
cagguucgagaagaggcaggc	5	0	LT-1
cagguucgagaagaggcaggc	6	0	LT-3
cagguucgagaagaggcaggc	1	0	JCK-2
cagguucgagaagaNgcaggc	1	1	LCK-1
cagguucgagaagaggcaggc	28	0	LCK-2
cagguucgagaagaggcaggc	15	0	LCK-3
cagguucgagaagaggcaggc	2	0	LT-2
cagguucgagaagaggcaggc	26	0	LCK-1
cagguucgagaagaggcaggca	1	0	LT-1
cagguucgagaagaggcaggca	1	0	JT-2
cagguucgagaagaggcaggca	7	0	JT-3
cagguucgagaagaggcaggca	12	0	LCK-1
cagguucgagaagaggcaggca	31	0	LCK-2
cagguucgagaagaggcaggca	2	0	LT-3
cagguucgagaagaAgcaggca	1	1	LT-3
cagguucgagaagaggcaggca	1	0	JCK-3
cagguucgagaagaggcaggcac	1	0	LT-2
cagguucgagaagaggcaggcac	2	0	JCK-1
cagguucgagaagaggcaggcac	2	0	LT-1
cagguucgagaagaggcaggcac	3	0	JCK-2
agguucgagaagaggcag	2	0	LCK-3
agguucgagaagaggcag	1	0	LT-1
agguucgagaagaggcagg	3	0	LT-2
agguucgagaagaggcagg	7	0	LT-3
agguucgagaagaggcagg	1	0	LT-1
agguucgagaagaggcagg	3	0	JT-2
agguucgagaagaggcagg	8	0	JT-3
agguucgagaagaggcaggc	2	0	LT-3
agguucgagaagaggcaggc	2	0	LT-2
agguucgagaagaggcaggc	1	0	JCK-2
agguucgagaagaggcaggc	2	0	LT-1
agguucgagaagaggcaggca	2	0	LT-3
agguucgagaagaggcaggcac	1	0	LT-1
gguucgagaagaggcagg	5	0	LCK-3
gguucgagaagaggcaggc	2	0	LT-2
gguucgagaCgaggcaggc	1	1	LT-2
gguucgagaagaggcaggc	1	0	LT-1
gguucgagaagaggcaggca	1	0	LT-1
guucgagaagaggcaggc	1	0	LT-3
guucgagaagaggcaggc	1	0	LT-1
guucgagaagaggcaggca	3	0	JT-3
guucgagaagaggcaggca	1	0	JT-2

Star Mature

aguuuuaggucagagaguagaacuuaaaaaacaauaagauaagggcaaaauauuggucuaagugaccaauuuuuuggaguuuguacuacauaaugagaccuuucgguaauuuuuauaaaaggagucaucauauaggacacaagaaaaucuaccagguggcccaaccuucuaguccuguggucaaguggaugaguuguguccaccauguuacagguucgagaagaggcaggcacaaacaaaacaaacaacc

...uucgagaagaggcaggca..................	2	0	LT-3
...uucgagaagaggcaggca..................	3	0	JT-3
...uucgagaagaggcaggca..................	4	0	JT-2
...uucgagaagaggcaggcac................	2	0	LT-2
...cgagaagaggcaggcacaaa..............	1	0	JCK-2
...cgagaagaggcaggcacaaacaaaaAa.......	4	1	JCK-3
...gagaagaggcaggcacaaaca............	1	0	JCK-2
...gagaagaggcaggcacaaaca............	1	0	JCK-3
...gagaagaggcaggcacaaacaaaU.........	1	1	JCK-2
...gagaagaggcaggcacaaacaaaU.........	1	1	JCK-3
...gagaagaggcaggcacaaacaaaU.........	2	1	JCK-1
...gagaagaggcaggcacaaacaaaacaaaca...	1	0	JT-2
...gagaagaggcaggcacaaacaaaacaaaca...	2	0	JT-3
...agaagaggcaggcacaaac............	1	0	JCK-3
...agaagaggcaggcacaaacaa..........	2	0	JT-2
...agaagaggcaggcacaaacaa..........	1	0	JT-3
...agaagaggcaggcacaaacaaa.........	3	0	JT-2
...agaagaggcaggcacaaacaaa.........	4	0	JT-3
...agaagaggcaggcacaaacaaaaca.......	1	0	JT-2
...agaagaggcaggcacaaacaaaaca.......	4	0	JT-3
...agaagaggcaggcacaaacaGaacaa......	1	1	JT-2
...agaagaggcaggcacaaacaaaacaa......	4	0	JT-3
...agaagaggcaggcacaaacaaaacaa......	1	0	JT-2
...agaagaggcaggcacaaacaaaacaaaca...	5	0	JCK-3
...agaagaggcaggcacaaacaaaacaaaca...	6	0	JCK-2
...agaagaggcaggcacaaacaaaacaaacaG..	1	1	JCK-2
...gaagaggcaggcacaaacaaaac........	1	0	JT-2
...gaagaggcaggcacaaacaaaac........	2	0	JT-3
...gaagaggcaggcacaaacaaaacaaaca...	1	0	JT-1
...gaagaggcaggcacaaacaaaacaaacaGc.	1	1	JT-2
...gaagaggcaggcacaaacaaaacaaacaGc.	2	1	JT-3
...aagaggcaggcacaaaca...........	1	0	JCK-1
...aagaggcaggcacaaacaaU..........	1	1	JCK-2
...aagaggcaggcacaaacaaaac........	2	0	JCK-1
...aagaggcaggcacaaacaaaacaa......	1	0	JCK-2
...aagaggcaggcacaaacaaaacaaac....	1	0	JT-2
...aagaggcaggcacaaacaaaacaaac....	2	0	JT-3
...aagaggcaggcacaaacaaaacaaac....	1	0	JCK-2
...aagaggcaggcacaaacaaaacaaac....	1	0	JCK-3
...aagaggcaggcacaaacaaaacaaacaG..	3	1	JT-2
...aagaggcaggcacaaacaaaacaaacaGcc	1	1	JCK-2
...agaggcaggcacaaacaaa..........	4	0	JT-2
...agaggcaggcacaaacaaa..........	4	0	JT-3
...agaggcaggcacaaacaaaa.........	1	0	JCK-3
...agaggcaggcacaaacaaaacaaa.....	10	0	JT-3
...agaggcaggcacaaacaaaacaaa.....	7	0	JT-2
...agaggcaggcacaaacaaaacaaaca...	2	0	JT-3
...agaggcaggcacaaacaaaacaaacaGc.	1	1	JCK-1
...agaggcaggcacaaacaaaacaaacaGc.	1	1	JCK-2
...gaggcaggcacaaacaaaaca.......	2	0	JT-2
...gCggcaggcacaaacaaaaca.......	1	1	JT-3
...gaggcaggcacaaacaaaaca.......	12	0	JT-3
...gaggcaggcacaaacaaaacaa......	5	0	JT-2
...gaggcaggcacaaacaaaacaa......	4	0	JT-3
...gaggcaggcacaaacaaaacaaa.....	5	0	JT-3
...gaggcaggcacaaacaaaacaaaca...	2	0	JT-3
...gaggcaggcacaaacaaaacaaaca...	1	0	JCK-3
...gaggcaggcacaaacaaaacaaaca...	1	0	JCK-2
...gaggcaggcacaaacaaaacaaaca...	3	0	JT-2
...gaggcaggcacaaacaaaacaaacaGc.	1	1	JCK-3
...gaggcaggcacaaacaaaacaaacaGc.	2	1	JCK-2
...gaggcaggcacaaacaaaacaaacaGc.	1	1	JCK-1
...aggcaggcacaaacaaaacaa......	7	0	JT-3
...aggcaggcacaaacaaaacaa......	3	0	JT-2
...aggcaggcacaaacaaaacaaac....	1	0	JCK-1
...aAgcaggcacaaacaaaacaaac....	1	1	JT-3
...aggcaggcacaaacaaaacaaac....	1	0	JT-2
...aggcaggcacaaacaaaacaaac....	3	0	JT-3
...aggcaggcacaaacaaaacaaac....	1	0	JCK-2
...aggcaggcacaaacaaaacaaaca...	1	0	JT-3

```
                                                                                                                                                                                      Star                                           Mature
aguuuuaggucagagaguagaacuuaaaaaacaauaagauaagggcaaaauauuggucuaagugaccaauuuuuuggaguuuguacuacauaaugagaccuuucgguaauuuuuauaaaaggagucaucauauaggacacaagaaaaucuaccagguggcccaaccuucuaguccuguggucaaguggaugaguuguguccaccauguuacagguucgagaagaggcaggcacaaacaaaacaaacaacc
...............................................................................................................................................................................................................................aggcaggcacaaCcaaaacaaaca...   1   1   JT-3
...............................................................................................................................................................................................................................aggcaggcacaaacaaaacaaacaGcc   1   1   JCK-2
...............................................................................................................................................................................................................................aggcaggcacaaacaaaacaaacaGcc   1   1   JCK-3
..................................................................................................................................................................................................................................caggcacaaacaaaacaaaU....   1   1   JCK-1
..................................................................................................................................................................................................................................caggcacaaacaaaacaaacU...   1   1   JCK-2
..................................................................................................................................................................................................................................caggcacaaacaaaacaaaca...   3   0   JT-2
..................................................................................................................................................................................................................................caggcacaaacaaaacaaacU...   3   1   JCK-1
..................................................................................................................................................................................................................................caggcacaaacaaaacaaaca...   7   0   JT-3
..................................................................................................................................................................................................................................caggcacaaacaaaacaaacaGc.   5   1   JT-3
..................................................................................................................................................................................................................................caggcacaaacaaaacaaacaGc.   7   1   JT-2
..................................................................................................................................................................................................................................caggcacaaacaaaacaaacaGc.   4   1   JCK-2
...................................................................................................................................................................................................................................aggcacaaacaaaacaaaca...   1   0   JCK-2
...................................................................................................................................................................................................................................aggcacaaacaaaacaaacaGc.   2   1   JCK-2
...................................................................................................................................................................................................................................aggcacaaacaaaacaaacaGc.   1   1   JCK-3
```
